# Host–virus resilience networks and viral inflammaging circuits in aging and long COVID: a CMV-centered perspective

**DOI:** 10.3389/fcimb.2026.1896480

**Published:** 2026-08-05

**Authors:** Ludmila Müller, Svetlana Di Benedetto, Viktor Müller

**Affiliations:** Max Planck Institute for Human Development, Center for Lifespan Psychology, Berlin, Germany

**Keywords:** aging, cytomegalovirus, host-virus resilience networks, immunosenescence, long covid, systems gerovirology, viral inflammaging circuits

## Abstract

Aging and long COVID are increasingly recognized as states of disrupted host homeostasis characterized by chronic inflammation, immune remodeling, metabolic dysfunction, and impaired stress adaptation. These alterations may compromise the mechanisms that normally maintain viral latency, thereby increasing susceptibility to reactivation of persistent viruses such as Cytomegalovirus (CMV). In this mini-review, we propose a systems-level framework in which latent viral reactivation emerges as a manifestation of declining organismal resilience rather than an isolated virological event. We introduce the concept of *host–virus resilience networks*, encompassing interconnected immune, metabolic, epigenetic, and cellular stress-response pathways that collectively preserve CMV latency across the lifespan. Age-associated immunosenescence, inflammaging, mitochondrial dysfunction, and epigenetic drift may progressively destabilize these networks, weakening antiviral surveillance and facilitating viral reactivation. We further propose the concept of *viral inflammaging circuits*, defined as self-reinforcing feedback loops in which chronic inflammation promotes viral reactivation, while viral activity further amplifies immune dysregulation, tissue stress, and inflammatory signaling. Within this framework, CMV is considered both a marker and a potential driver of immune aging through persistent antigenic stimulation, T-cell remodeling, and chronic inflammatory activation. Long COVID may represent a convergent resilience failure state in which persistent immune perturbation and metabolic stress intersect with latent herpesvirus biology. By integrating concepts from geroscience, immunology, and systems virology, this review aims to provide a conceptual model linking CMV persistence to network-level dysregulation in ageing and post-viral syndromes and highlights the importance of resilience-based approaches for understanding chronic inflammatory disease progression across the lifespan.

## 1 Introduction

A large proportion of the human population harbors latent viruses that persist throughout life in a state of long-term host–virus coexistence (Nikolich-Zugich et al., 2020). Among these, herpesviruses establish lifelong persistence through highly regulated latency programs that minimize viral gene expression while evading immune elimination. Traditionally, viral latency has often been viewed as a largely passive dormant state interrupted only by episodic reactivation. However, growing evidence suggests that latent viral persistence represents a dynamic equilibrium actively maintained by interconnected immune, metabolic, epigenetic, and cellular stress-response mechanisms (Maguire et al., 2024). In this context, the maintenance of latency depends not solely on antiviral immunity, but on the integrity of broader host homeostatic systems that continuously constrain viral activity while preserving tissue function and immune balance (Klenerman and Oxenius, 2016; Müller and Di Benedetto, 2024a).

Aging profoundly alters these regulatory networks. Hallmarks of aging, including immunosenescence, inflammaging, mitochondrial dysfunction, genomic instability, and epigenetic drift, collectively impair the capacity of the host to maintain physiological resilience (Liu et al., 2013; Müller et al., 2017; Pawelec, 2018). Similar alterations have also been described in long COVID, a heterogeneous post-viral condition associated with persistent immune activation, metabolic disturbances, endothelial dysfunction, and chronic inflammatory signaling. Although aging and long COVID differ in origin and timescale, both may represent states of disrupted host resilience in which the mechanisms that normally preserve viral latency become progressively destabilized (Davis et al., 2023).

Cytomegalovirus represents a paradigmatic latent virus within this framework. CMV infection is highly prevalent worldwide and exerts profound effects on immune organization across the lifespan (Di Benedetto et al., 2015). Consequently, CMV has emerged not only as a marker of immune aging, but also as a potential contributor to inflammaging and age-associated tissue vulnerability (Di Benedetto et al., 2019a; Heath and Grant, 2020). In long COVID, persistent immune dysregulation and cellular stress may further intersect with latent CMV biology, potentially facilitating viral reactivation or amplifying chronic inflammatory states (Müller and Di Benedetto, 2024a; Maguire et al., 2024).

Although this review adopts a CMV-centered perspective, the conceptual framework of host–virus resilience networks and viral inflammaging circuits may extend to other latent herpesviruses, including Epstein–Barr virus (EBV) and varicella-zoster virus (VZV). Similar to CMV, reactivation of these viruses has been associated with aging-associated immune dysfunction and has been implicated in subsets of patients with long COVID (Bassyouni et al., 2019; Gold et al., 2021; Simonnet et al., 2021; Müller and Di Benedetto, 2023a; Peluso et al., 2023; Hakami et al., 2024). Nevertheless, CMV provides a particularly informative model because of its unique capacity to drive lifelong memory inflation, extensive T-cell repertoire remodeling, and systemic immune adaptation. Consequently, CMV represents an ideal paradigm for investigating how chronic viral persistence interacts with biological aging to reshape host resilience networks.

In this mini-review, we propose two complementary conceptual frameworks to describe these processes. First, we introduce the concept of *host–virus resilience networks*, defined as interconnected immune, metabolic, epigenetic, and stress-response systems that collectively maintain viral latency and host homeostasis across the lifespan through dynamic bidirectional interactions between host regulatory mechanisms and latent viral programs. Second, we propose the concept of *viral inflammaging circuits*, referring to self-reinforcing feedback loops in which chronic inflammation promotes viral reactivation, while viral activity further amplifies immune dysregulation, tissue stress, and inflammatory signaling. Together, these frameworks provide a systems-level perspective linking CMV persistence to network-wide dysregulation in aging and long COVID and may help explain how latent viral reactivation contributes to chronic inflammatory and degenerative disease processes.

## 2 Host–virus resilience networks: maintaining CMV latency

**Figure 1:**
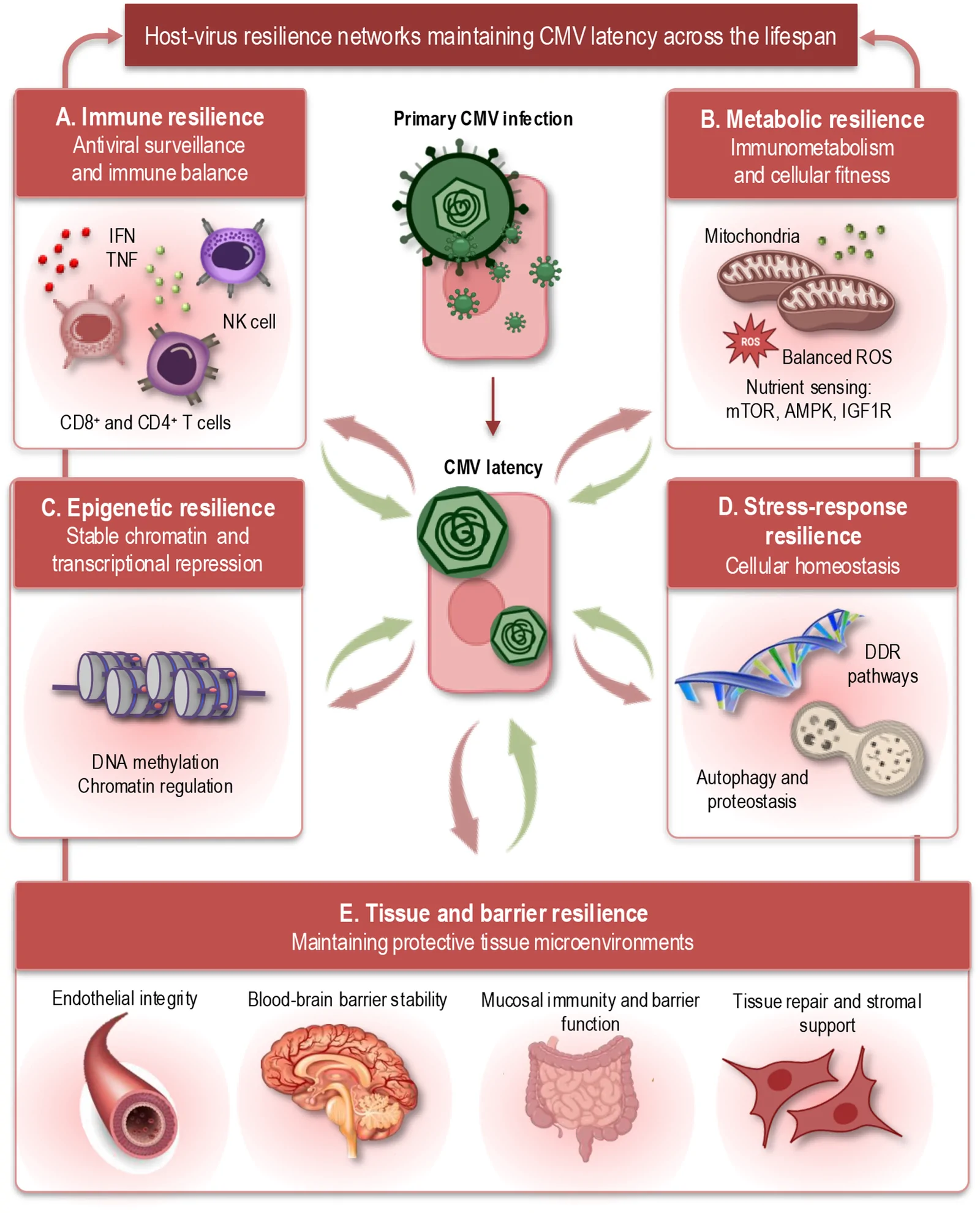
Schematic representation of the interconnected *host–virus resilience networks* that actively maintain latent CMV infection under physiological conditions. Under physiological conditions, CMV latency is sustained through dynamic and bidirectional interactions between host regulatory systems (green arrows) and latent viral programs (red arrows). **(A)** Immune resilience encompasses antiviral surveillance mediated by CD8^+^ and CD4^+^ T cells, NK cells, interferon signaling, and immune memory, which collectively constrain viral reactivation while preserving immune homeostasis. **(B)** Metabolic resilience supports antiviral function through mitochondrial fitness, balanced reactive oxygen species (ROS) signaling, metabolic flexibility, and nutrient-sensing pathways, including mTOR, AMPK, and IGF1R. **(C)** Epigenetic resilience maintains transcriptional repression of latent viral genomes through chromatin regulation, DNA methylation, and other epigenetic mechanisms. **(D)** Cellular stress-response resilience preserves homeostasis through DNA damage response (DDR) pathways, proteostasis, and autophagy, thereby limiting cellular conditions that favor viral reactivation. **(E)** Tissue and barrier resilience provides an additional layer of control through endothelial integrity, blood–brain barrier stability, mucosal immunity, and stromal support networks that maintain protective tissue microenvironments, regulate immune-cell trafficking, and restrict inflammatory dissemination. Bidirectional interactions among these interconnected resilience domains collectively preserve host–virus equilibrium, maintain CMV latency, and support tissue homeostasis across the lifespan.

The persistence of latent CMV throughout life reflects a highly coordinated state of host–virus equilibrium maintained by interconnected biological systems operating across molecular, cellular, and tissue levels. Rather than representing a passive inactive state, CMV latency is increasingly recognized as a dynamic and actively regulated condition requiring continuous host surveillance and adaptive regulation (Goodrum et al., 2012; Müller and Di Benedetto, 2024a). In this section we examine in greater detail the concept of *host–virus resilience networks* that can be defined as integrated immune, metabolic, epigenetic, stress-response, and tissue-support systems that collectively sustain viral latency while preserving tissue homeostasis and organismal stability under physiological stress. In other words, such bidirectional, multiscale interactions between host regulatory systems and latent viral programs can collectively maintain—or, under conditions of resilience failure, destabilize—the equilibrium between viral persistence and host physiological homeostasis. These networks may function cooperatively to constrain viral transcription, prevent productive reactivation, and minimize inflammatory tissue damage despite lifelong viral persistence (**Figure 1**).

### 2.1 Integrated immune–metabolic control of latency

Immune surveillance represents a central component of host–virus resilience networks (**Figure 1A**). CMV latency is continuously controlled by coordinated innate and adaptive immune responses that suppress viral gene expression and eliminate cells undergoing productive viral activation. Among these mechanisms, CMV-specific CD8^+^ cytotoxic T lymphocytes play a major role in maintaining latency through recognition and elimination of infected cells, as well as through secretion of antiviral cytokines, including interferon-γ (IFN-γ) and tumor necrosis factor-α (TNF-α). Natural killer (NK) cells provide an additional layer of control by recognizing stress-induced ligands and altered major histocompatibility complex expression associated with viral infection. Interferon signaling further contributes to viral repression by inducing broad antiviral transcriptional programs and regulating immune-cell communication within infected tissues (Hazeldine and Lord, 2013; Di Benedetto et al., 2019a; Lu et al., 2022; Chaudhry et al., 2024).

Unlike acute viral infections, CMV establishes lifelong antigenic persistence that requires durable immune adaptation over decades. This persistent host–virus interaction is associated with extensive remodeling of immune architecture, including maintenance of large populations of CMV-specific memory T cells (Di Benedetto et al., 2015; Müller et al., 2017). A characteristic feature of CMV infection is “memory inflation,” in which virus-specific T-cell populations progressively accumulate and remain functionally active throughout life (Griessl et al., 2021; Alonso-Arias et al., 2022). Although these long-lived immune populations contribute to viral control, they also reflect the substantial energetic and regulatory burden imposed by lifelong CMV surveillance. Maintenance of immune memory therefore represents not only an immunological process, but also a metabolically demanding adaptive state requiring continuous coordination between immune signaling and cellular bioenergetics.

Metabolic resilience (**Figure 1B**) is tightly integrated with antiviral immune function. Effective immune surveillance depends on mitochondrial fitness, metabolic flexibility, and precise regulation of cellular energy utilization (Marques et al., 2024). Mitochondria support immune-cell activation and effector responses through ATP generation, redox regulation, calcium signaling, and biosynthetic adaptation. Beyond their bioenergetic role, mitochondria also function as central hubs of innate antiviral immunity by coordinating mitochondrial antiviral signaling (MAVS), interferon induction, inflammasome activation, and cellular stress responses (Soderberg-Naucler, 2006; Muri and Kopf, 2021; Marques et al., 2024; Mukherjee et al., 2024).

Emerging evidence indicates that CMV actively interacts with mitochondrial networks during both productive infection and latency, modulating mitochondrial dynamics, metabolism, calcium homeostasis, and redox balance to create a cellular environment that favors viral persistence while limiting excessive antiviral responses. Balanced production of reactive oxygen species (ROS) contributes to intracellular signaling and antimicrobial defense, whereas tightly regulated nutrient-sensing pathways coordinate transitions between anabolic growth and stress-adaptive states (Soderberg-Naucler, 2006; Combs et al., 2020). Together, these observations suggest that metabolic resilience reflects not only the ability of host cells to sustain efficient immune function but also their capacity to preserve mitochondrial homeostasis despite continuous bidirectional interactions with latent viral programs.

Pathways involving mechanistic target of rapamycin (mTOR), AMP-activated protein kinase (AMPK), and insulin-like growth factor 1 receptor (IGF1R) signaling are particularly important in coordinating immune metabolism with cellular stress responses and viral persistence. mTOR signaling can support T-cell activation, differentiation, and protein synthesis, while also influencing CMV replication and latency-associated transcriptional programs (Gabriel et al., 2021). CMV itself can directly manipulate host metabolic pathways, including AMPK-associated glycolytic activation, to support viral replication and persistence (Dunn et al., 2021). IGF1R signaling may further integrate nutrient availability and growth-related signaling with immune regulation and tissue maintenance (Khan et al., 2025). Together, these interconnected metabolic pathways can help maintain intracellular conditions compatible with antiviral resilience and stable viral repression. Thus, immune and metabolic homeostasis should not be considered independent processes, but rather integrated components of broader host–virus resilience networks that sustain CMV latency across the lifespan.

### 2.2 Epigenetic and cellular stress control of viral repression

A defining feature of CMV latency is the epigenetic silencing of viral genomes within host cells (**Figure 1C**). Following infection, latent CMV genomes persist as chromatinized episomes that become integrated into host chromatin regulatory environments (Liu et al., 2013). The maintenance of latency therefore depends on active transcriptional repression mediated by histone modifications, chromatin compaction, DNA methylation, and recruitment of host epigenetic regulatory complexes. In this context, latency represents a continuously maintained epigenetic state rather than complete viral inactivity.

Chromatin organization plays a particularly important role in restricting viral immediate-early gene expression, which is required for productive viral replication. Repressive histone marks and heterochromatin-associated proteins contribute to silencing of viral promoters, whereas permissive chromatin remodeling may facilitate viral transcriptional activation under conditions of cellular stress. Host epigenetic machinery may therefore act as a critical interface between environmental signals, cellular physiology, and viral transcriptional control (Goodrum et al., 2012; Liu et al., 2013; Cevenini et al., 2025).

Cellular stress-response pathways are similarly integrated into the regulation of viral latency (**Figure 1D**). DNA damage response (DDR) signaling pathways, including ATM- and ATR-mediated signaling, can contribute to preservation of genomic stability and coordination of cellular adaptation to oxidative or replicative stress. These pathways may additionally influence viral transcription through modulation of chromatin accessibility, transcription-factor activity, and stress-responsive signaling cascades (Goodrum et al., 2012). Autophagy and proteostasis networks further support resilience by maintaining intracellular quality control through removal of damaged proteins and dysfunctional organelles. Beyond their homeostatic functions, these pathways may participate in immune regulation and antiviral defense by influencing antigen presentation, inflammatory signaling, and cellular stress adaptation (Romao and Munz, 2011; Konig et al., 2021; Gomez-Virgilio et al., 2022).

Collectively, these observations suggest that CMV latency appears to be embedded within broader host regulatory systems governing chromatin integrity, stress adaptation, and intracellular homeostasis. Epigenetic regulation and cellular stress responses may therefore represent essential components of host–virus resilience networks that continuously suppress viral activation while preserving cellular function.

### 2.3 Tissue and barrier resilience

Host–virus resilience networks extend beyond intracellular and immune regulatory systems to include tissue-level and barrier mechanisms that preserve compartmental integrity and regulate local immune environments (**Figure 1E**). Endothelial barriers, mucosal surfaces, stromal support networks, and specialized tissue interfaces contribute to the maintenance of viral latency by limiting inflammatory dissemination, regulating immune-cell trafficking, and sustaining tissue-specific homeostasis.

Endothelial integrity plays a central role in host–virus resilience because endothelial cells not only regulate vascular homeostasis but may also serve as targets and reservoirs of latent CMV persistence (Dupont and Reeves, 2016). As key components of the vascular niche, endothelial cells coordinate leukocyte trafficking, cytokine signaling, coagulation pathways, and vascular permeability, thereby maintaining balanced tissue immune surveillance while limiting excessive inflammatory activation (Ungvari et al., 2018; Bomfim et al., 2023). Disruption of endothelial homeostasis, which becomes increasingly common with aging and chronic inflammatory conditions, may therefore facilitate both inflammatory amplification and viral reactivation by promoting immune-cell infiltration and local tissue injury.

The blood–brain barrier (BBB) represents a highly specialized extension of tissue resilience, integrating endothelial cells, astrocytes, pericytes, and extracellular matrix components within the neurovascular unit to preserve central nervous system homeostasis. Beyond restricting peripheral immune-cell infiltration, the BBB actively regulates molecular transport, neuroimmune communication, and inflammatory signaling between the circulation and the brain. Experimental and clinical evidence suggests that CMV can infect endothelial cells and glial populations, while BBB dysfunction may increase susceptibility to persistent neuroinflammation by facilitating inflammatory cell recruitment and disrupting local immune regulation (Montagne et al., 2015; Kadry et al., 2020). Preservation of BBB integrity is therefore likely to represent an essential component of host–virus resilience, limiting the propagation of systemic inflammatory signals into the brain and reducing the risk of chronic neuroinflammatory sequelae associated with aging and long COVID.

Mucosal immunity represents another critical layer of resilience because mucosal tissues serve as major interfaces between the host and external environment (Kernbauer et al., 2014; Zhou et al., 2025). Coordinated interactions among epithelial cells, resident immune populations, stromal cells, and local microbiota help maintain immune tolerance while preserving antimicrobial defense (Belkaid and Harrison, 2017; Müller and Di Benedetto, 2025a). Stromal support networks further contribute to tissue resilience through regulation of extracellular matrix organization, immune-cell positioning, tissue repair, and intercellular signaling. Together, these tissue-level systems create local microenvironments that can support balanced immune regulation and constrain inflammatory amplification associated with latent viral persistence (Alexandre and Mueller, 2018; Krausgruber et al., 2020).

Viewed collectively, host–virus resilience networks encompass far more than antiviral immune surveillance alone. Instead, they represent multilayered systems integrating immune regulation, metabolic homeostasis, epigenetic stability, stress adaptation, and tissue integrity to preserve long-term equilibrium between host physiology and latent viral persistence.

## 3 Breakdown of resilience in aging and long COVID

**Figure 2:**
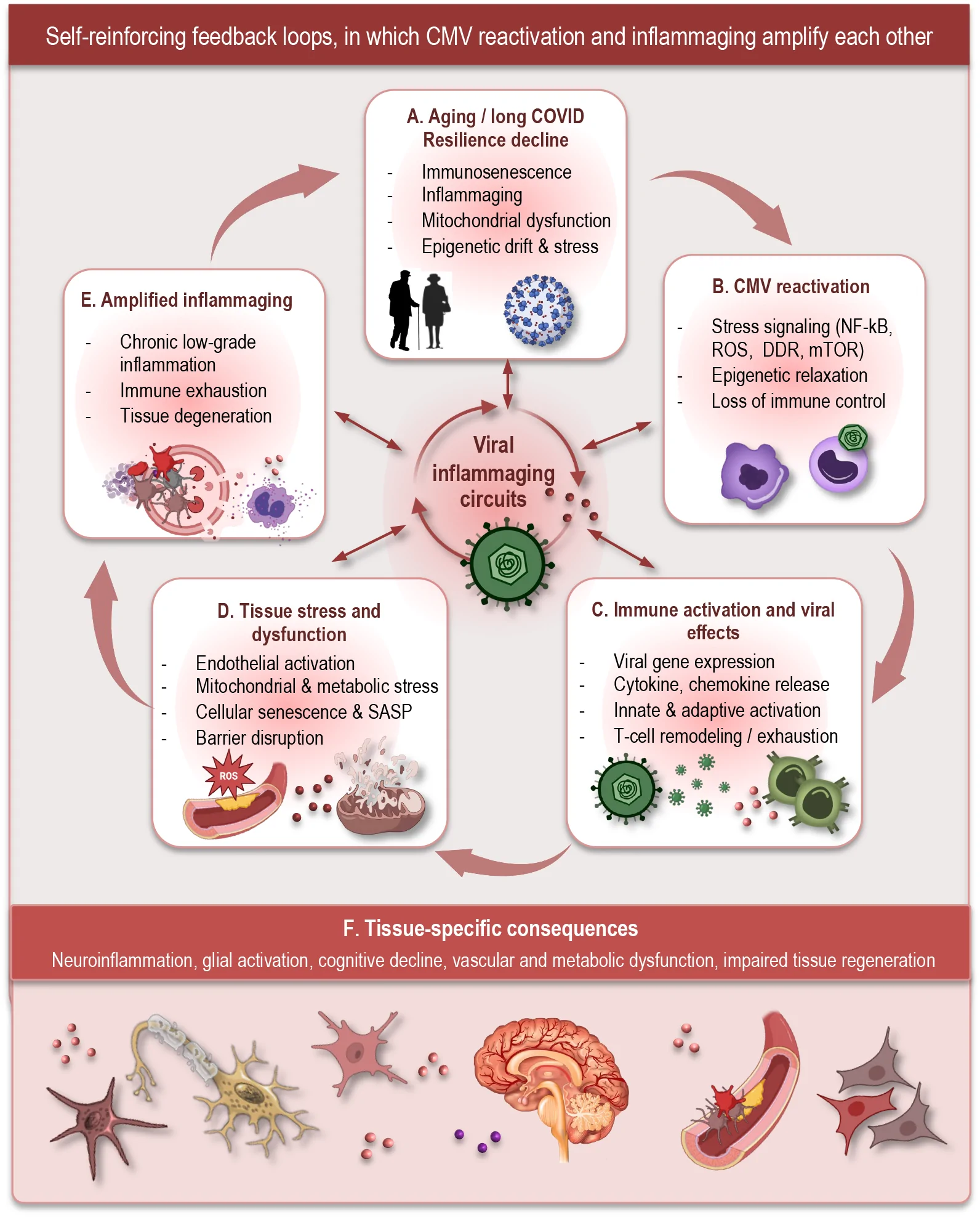
Schematic illustration of the proposed *viral inflammaging circuits*, in which declining host resilience promotes recurrent CMV reactivation and chronic inflammatory remodeling. **(A)** Aging and long COVID weaken host resilience networks through immunosenescence, inflammaging, mitochondrial dysfunction, and epigenetic stress. **(B)** These changes increase susceptibility to CMV reactivation via activation of stress-responsive pathways, including NF-κB, ROS, DNA damage response (DDR), mTOR signaling, and epigenetic derepression. **(C)** Viral reactivation induces viral gene expression, cytokine and chemokine production, and sustained innate and adaptive immune activation, resulting in chronic antigenic stimulation and immune-cell remodeling. **(D)** Persistent immune activation promotes tissue dysfunction, including endothelial activation, metabolic impairment, cellular senescence with SASP, and barrier disruption. **(E)** These alterations further amplify chronic inflammation, immune dysfunction, and tissue degeneration, thereby reinforcing conditions that favor subsequent viral reactivation. Together, these processes form self-sustaining *viral inflammaging circuits* that perpetuate pathological host–virus interactions. **(F)** The cumulative consequences include neuroinflammation, cognitive decline, vascular and metabolic dysfunction, impaired tissue regeneration, and progressive loss of tissue homeostasis. Bidirectional interactions between host resilience mechanisms and latent viral activity are indicated throughout the network.

Although host–virus resilience networks support long-term control of latent CMV under physiological conditions, these systems undergo progressive destabilization during aging and chronic inflammatory disease states (Müller and Di Benedetto, 2024a). Aging is associated with cumulative molecular and cellular alterations that impair immune coordination, metabolic flexibility, stress adaptation, and tissue homeostasis (**Figure 2**). Similar disturbances have also been observed in long COVID, suggesting that both conditions may converge on common pathways of resilience decline (Akbar and Gilroy, 2020; Müller and Di Benedetto, 2024b; Peluso and Deeks, 2024). As these interconnected regulatory networks deteriorate, the capacity of the host to maintain stable viral repression may progressively weaken, thereby increasing susceptibility to latent viral reactivation and chronic inflammatory amplification (Akbar and Gilroy, 2020; Lord et al., 2024; Müller and Di Benedetto, 2024a; Phetsouphanh et al., 2024).

### 3.1 Immunosenescence and inflammaging

Immunosenescence and inflammaging represent central mechanisms contributing to the breakdown of host–virus resilience networks during aging (Di Benedetto et al., 2019b). Rather than reflecting simple immune decline, immunosenescence involves extensive remodeling of innate and adaptive immune systems that alters immune composition, functionality, and regulatory balance. These changes include thymic involution, reduced naïve T-cell output, contraction of T-cell receptor diversity, impaired antigen responsiveness, and accumulation of highly differentiated memory and effector lymphocyte populations. Together, these alterations may reduce immune adaptability and compromise the capacity to maintain efficient antiviral surveillance under conditions of physiological stress (Müller et al., 2019; Müller and Di Benedetto, 2024a).

Both CD8^+^ and CD4^+^ T-cell compartments play important roles in these processes. Although CMV-associated immune remodeling is often discussed primarily in the context of expanded CD8^+^ cytotoxic T-cell populations, CD4^+^ T cells are also critical regulators of antiviral resilience (Di Benedetto et al., 2015). CMV-specific CD4^+^ T cells support antiviral immunity through cytokine production, maintenance of CD8^+^ T-cell function, orchestration of B-cell responses, and regulation of tissue immune environments (Di Benedetto et al., 2019a; Müller and Di Benedetto, 2026). During aging, however, CD4^+^ T-cell populations undergo profound functional changes, including reduced proliferative capacity, impaired helper function, altered cytokine secretion profiles, and increased susceptibility to senescence and exhaustion. Expansion of highly differentiated CD4^+^CD28^+^ T-cell populations, frequently associated with chronic CMV infection, has been linked to pro-inflammatory phenotypes and vascular pathology. Consequently, age-associated dysfunction within both CD4^+^ and CD8^+^ T-cell compartments may weaken coordinated antiviral immunity and destabilize host–virus equilibrium (Barnes et al., 2015; Lu et al., 2022; Chaudhry et al., 2024; Müller and Di Benedetto, 2024a).

In addition to age-associated immune remodeling, host-specific factors—including impaired type I interferon responses resulting from genetic defects or neutralizing autoantibodies—may further compromise antiviral resilience and increase susceptibility to viral reactivation, thereby contributing to interindividual variability in CMV control during aging and long COVID (Bastard et al., 2020; Casanova and Anderson, 2023).

Persistent CMV infection exerts a particularly strong influence on immune aging because lifelong antigenic stimulation continuously engages immune regulatory systems. CMV-associated immune remodeling is characterized by progressive expansion and long-term maintenance of virus-specific T-cell populations (Di Benedetto et al., 2015). Although these cells contribute to viral control, their accumulation may occur at the expense of immune repertoire diversity and adaptive flexibility. Over time, chronic antigenic stimulation may promote immune exhaustion and senescence, reflected by impaired proliferative responses, altered effector function, and expression of inhibitory receptors associated with diminished immune competence (Dupont and Reeves, 2016; Alonso-Arias et al., 2022; Müller and Di Benedetto, 2024a).

Importantly, the impact of CMV on immune aging is influenced not only by lifelong viral persistence but also by the characteristics of the initial host–virus encounter. Experimental studies in murine CMV infection have demonstrated that higher infectious doses induce more pronounced memory inflation and expansion of effector-memory CD8^+^ T cells, resulting in accelerated immune remodeling and impaired responses to heterologous viral infections later in life (Redeker et al., 2017). These findings suggest that host–virus resilience is shaped not simply by CMV seropositivity but also by the magnitude and quality of antiviral immune responses established early during infection, thereby providing one potential explanation for interindividual variability in immune aging and susceptibility to age-related disease.

In parallel with adaptive immune remodeling, aging is associated with inflammaging. This persistent inflammatory state is characterized by increased systemic levels of inflammatory mediators, including interleukin-6 (IL-6), TNF-α, C-reactive protein, and interferon-associated cytokines (Franceschi and Campisi, 2014; Di Benedetto et al., 2019a; Thomas et al., 2020). Multiple mechanisms contribute to inflammaging, including mitochondrial dysfunction, oxidative stress, impaired autophagy, innate immune activation, and accumulation of senescent cells exhibiting the senescence-associated secretory phenotype (SASP). SASP-associated secretion of cytokines, chemokines, proteases, and growth factors may further amplify tissue inflammation and alter local immune microenvironments (Coppe et al., 2010; Meier et al., 2023; Song et al., 2023; Soraci et al., 2024; Müller and Di Benedetto, 2025b; Tamatta et al., 2025).

Importantly, immunosenescence and inflammaging are tightly interconnected rather than independent processes. Chronic inflammatory signaling may impair antiviral immune responses by promoting T-cell dysfunction, altering interferon responsiveness, and disrupting immune-cell communication networks (Müller and Di Benedetto, 2024a; Müller and Di Benedetto, 2025b). At the same time, insufficient viral control may further sustain inflammatory activation through recurrent antigenic stimulation and low-level viral reactivation. Within this framework, CMV may function both as a driver and a consequence of immune aging, participating in bidirectional feedback loops that progressively erode host resilience and destabilize viral latency.

### 3.2 Metabolic and mitochondrial dysfunction

Mitochondrial dysfunction represents another central feature of aging-associated resilience decline and has profound implications for viral latency control. Mitochondria regulate not only cellular energy production but also redox balance, innate immune signaling, apoptosis, and inflammatory responses (Miwa et al., 2022; Lopez-Otin et al., 2023). Aging is associated with impaired oxidative phosphorylation, reduced mitochondrial quality control, accumulation of mitochondrial DNA damage, and increased production of ROS. Excessive ROS generation contributes to oxidative stress, macromolecular damage, and activation of pro-inflammatory signaling pathways that may favor viral reactivation (Marques et al., 2024; Mukherjee et al., 2024; Meichsner et al., 2026).

Altered immunometabolism further compromises antiviral defense mechanisms. Effective immune responses require coordinated metabolic adaptation to support immune-cell proliferation, cytokine production, and cytotoxic activity. However, aging-associated metabolic inflexibility may impair immune cell function while promoting chronic inflammatory activation (Lee et al., 2021; Muri and Kopf, 2021; Patel et al., 2022). Dysregulation of nutrient-sensing pathways, including mTOR, AMPK, and IGF1R signaling, may additionally disrupt the balance between anabolic and stress-adaptive programs that normally constrain viral activity (Franceschi et al., 2018; Marrella et al., 2022; Proal and VanElzakker, 2025; Müller et al., 2026a). Importantly, metabolic stress may directly influence viral transcriptional regulation by altering chromatin organization, redox-sensitive signaling pathways, and cellular stress responses linked to CMV reactivation.

Endothelial dysfunction may further exacerbate these processes. Aging-associated vascular inflammation and impaired endothelial integrity contribute to altered tissue perfusion, inflammatory cell recruitment, and systemic oxidative stress (Rahbar and Soderberg-Naucler, 2005; Kovacic et al., 2011). Because endothelial cells can serve as important reservoirs and targets of CMV infection, endothelial injury may create local microenvironments permissive for viral reactivation and inflammatory amplification (Rahbar and Soderberg-Naucler, 2005; Walker et al., 2009).

### 3.3 Long COVID as a state of impaired host resilience

Long COVID has emerged as a complex multisystem condition characterized by persistent symptoms and prolonged physiological dysregulation following acute severe acute respiratory syndrome coronavirus 2 (SARS-CoV-2) infection (Sherif et al., 2023). Although its underlying mechanisms remain incompletely understood, increasing evidence suggests that long COVID involves chronic disruption of immune, metabolic, and vascular homeostasis that overlaps substantially with biological processes associated with aging (Davis et al., 2023; Müller and Di Benedetto, 2023b). Persistent immune activation, altered cytokine profiles, and interferon dysregulation have been observed in subsets of patients months after acute infection, suggesting incomplete resolution of inflammatory responses (Alvarez-Heredia et al., 2023; Müller and Di Benedetto, 2023b; Lord et al., 2024).

In parallel, long COVID has been associated with neuroimmune alterations, endothelial injury, mitochondrial dysfunction, and chronic metabolic stress. These abnormalities may impair cellular stress adaptation and compromise the resilience networks that normally maintain latent viral repression (Müller and Di Benedetto, 2021; Müller and Di Benedetto, 2024a; Gaspar et al., 2025; Hein et al., 2025). Several studies have further reported associations between long COVID and reactivation of latent herpesviruses, including CMV and Epstein–Barr virus, although the causal significance of these findings remains under investigation (Gold et al., 2021; Soderberg-Naucler, 2021; Peluso et al., 2023; Vojdani et al., 2024; Chauhan et al., 2025; Gaspar et al., 2025). Rather than representing isolated virological events, such reactivation episodes may reflect broader failures of host resilience under conditions of sustained inflammatory and metabolic stress.

The degree of resilience impairment is likely to vary considerably among individuals. In addition to age-associated immunosenescence and chronic inflammatory remodeling, host-specific factors—including genetic variation, pre-existing immune status, and defects in interferon-mediated antiviral responses—may further influence the stability of host–virus resilience networks and susceptibility to latent viral reactivation. For example, neutralizing autoantibodies against type I interferons, which become more prevalent with advancing age, have been associated with severe COVID-19 and impaired antiviral immunity, providing one potential mechanism contributing to interindividual variability in CMV control and long COVID manifestations (Bastard et al., 2020; Casanova and Anderson, 2023).

Interindividual variability in host resilience likely reflects the combined influence of age, pre-existing immune status, viral coinfections, and host-specific immune determinants. Consistent with this concept, chronic viral coinfections have recently been shown to differentially influence the risk of developing long COVID, supporting the notion that latent viruses contribute to disease heterogeneity rather than acting as uniform pathogenic drivers (Peluso et al., 2023).

Conceptually, long COVID may therefore represent a convergent resilience failure state that may accelerate aspects of biological aging relevant to viral control (Müller and Di Benedetto, 2024b). Shared features, including immune exhaustion, mitochondrial dysfunction, chronic inflammation, and endothelial dysregulation, suggest that aging and long COVID may converge on common pathways that destabilize host–virus equilibrium and increase susceptibility to latent viral reactivation.

## 4 Viral inflammaging circuits: self-reinforcing feedback loops

The progressive deterioration of host–virus resilience networks during aging may establish conditions in which latent viral reactivation and chronic inflammation reinforce one another through self-sustaining feedback mechanisms (**Figure 2**). To conceptualize these multidirectional interactions, we propose the framework of *viral inflammaging circuits*, defined as interconnected feedback networks in which aging-associated inflammation and cellular stress facilitate viral reactivation, while viral activity further promotes chronic inflammation, immune dysfunction, and tissue degeneration. These circuits may operate across multiple physiological systems and contribute to the heterogeneity of aging trajectories and post-viral disease manifestations. Rather than separating viral persistence from broader aging biology, this framework positions latent viruses such as CMV as dynamic participants within chronic inflammatory ecosystems that evolve throughout the lifespan.

### 4.1 From viral latency to inflammatory amplification

As discussed in the preceding sections, the maintenance of CMV latency under physiological conditions relies on interconnected host–virus resilience networks that integrate immune surveillance, metabolic regulation, epigenetic control, cellular stress responses, and tissue homeostasis (Forte et al., 2020). However, aging-associated chronic inflammation may progressively destabilize these regulatory systems and create intracellular environments permissive for viral reactivation (**Figure 2A**). Elevated levels of pro-inflammatory cytokines, oxidative stress, mitochondrial dysfunction, and persistent activation of stress-responsive signaling pathways may collectively alter chromatin regulation and transcriptional control of latent viral genomes (Forte et al., 2018; Forte et al., 2020; Müller and Di Benedetto, 2024c).

In particular, inflammatory mediators such as TNF-α and IL-6, together with activation of NF-κB-associated pathways, have been implicated in the induction of viral immediate-early gene expression that initiates transition from latency toward productive viral activity (Forte et al., 2018). In addition, inflammatory IL-6 signaling has been shown to regulate CMV reactivation in dendritic cells through ERK–MAPK-associated pathways, further connecting inflammatory cytokine networks with viral latency control (Reeves and Compton, 2011).

Recent studies additionally indicate that CMV latency itself actively remodels inflammatory and interferon-responsive pathways within infected cells (**Figures 2B, C**). Proteomic analyses demonstrated that latent CMV infection suppresses interferon-inducible genes, including IFI16 and MHC class II-associated pathways, thereby modulating host inflammatory signaling and viral transcriptional regulation (Elder et al., 2019). Experimental studies further showed that activation of NF-κB-responsive enhancer elements can directly de-silence CMV immediate-early gene expression in quiescently infected cells, supporting the concept that inflammatory stress-responsive transcriptional programs are central regulators of viral reactivation (Liu et al., 2010).

Animal and translational studies also support the existence of chronic inflammatory feedback loops associated with CMV persistence. Murine CMV models demonstrated that latent infection drives recruitment and activation of CD4^+^ T-cell populations that can promote local viral reactivation and inflammatory amplification within tissues (Jackson et al., 2021). In humans, CMV seropositivity has been associated with elevated circulating inflammatory mediators, altered T-cell cytokine profiles, and cognitive decline in elderly populations, supporting the link between latent viral persistence and systemic inflammaging (Di Benedetto et al., 2019a). Recent work further highlights that CMV-associated immune remodeling contributes to chronic immune activation and immunosenescence during aging (Müller and Di Benedetto, 2024a). These findings collectively support the concept of *viral inflammaging circuits*, in which inflammatory stress promotes CMV reactivation, while recurrent viral activity further amplifies immune activation, tissue stress, and chronic inflammatory remodeling.

Cellular senescence may further amplify these processes (**Figures 2D, E**). Senescent cells accumulate during aging in response to DNA damage, mitochondrial dysfunction, telomere attrition, and chronic stress exposure. SASP-associated inflammatory signaling may promote viral reactivation directly through activation of stress-responsive transcriptional programs or indirectly by impairing local immune regulation and tissue homeostasis. At the same time, viral reactivation itself may enhance senescence-associated pathways by increasing oxidative stress, DNA damage signaling, and inflammatory cytokine production, thereby reinforcing inflammatory amplification (Müller et al., 2017; Forte et al., 2020; Müller and Di Benedetto, 2024a). In this context, chronic inflammatory activation and viral persistence become mutually reinforcing processes rather than separate pathological phenomena.

Importantly, viral inflammaging circuits likely operate across multiple biological scales, from intracellular signaling pathways to systemic inflammatory networks. At the molecular level, oxidative stress, mitochondrial dysfunction, impaired autophagy, and DNA damage response activation may influence viral transcriptional regulation (Xiaofei and Kowalik, 2014; Jackson, 2015; Forte et al., 2018). At the cellular level, chronic antigenic stimulation promotes immune-cell remodeling and senescence (Wertheimer et al., 2014; Di Benedetto et al., 2015; Nikolich-Žugich, 2018; Müller and Di Benedetto, 2024a). At the tissue level, endothelial dysfunction, stromal alterations, and barrier instability may facilitate inflammatory propagation and local viral activity (Rahbar and Soderberg-Naucler, 2005; Di Benedetto et al., 2019b; Forte et al., 2020; Müller and Di Benedetto, 2021).

Collectively, these processes may generate self-perpetuating cycles. Enhanced inflammaging increases the likelihood of further viral reactivation, while repeated viral activity contributes to progressive immune exhaustion and tissue dysfunction. Such bidirectional interactions may become particularly pronounced under conditions of impaired resilience, including advanced age and long COVID. Viral inflammaging circuits therefore provide a systems-level model linking latent viral persistence to chronic inflammatory and degenerative processes across multiple physiological systems.

### 4.2 Tissue-specific inflammaging circuits and their consequences

The consequences of viral inflammaging circuits are unlikely to be uniform across tissues and organs. Instead, tissue-specific vulnerability, regenerative capacity, local immune composition, and barrier integrity may determine how chronic inflammatory and viral stress responses manifest during aging and long COVID (**Figure 2F**). Because CMV persistence interacts with endothelial, immune, stromal, and metabolic regulatory systems throughout the body, viral inflammaging circuits may contribute to multisystem pathological remodeling rather than isolated organ dysfunction (Müller and Di Benedetto, 2024c).

Within the nervous system, chronic inflammatory activation may contribute to neuroinflammation, glial reactivity, and impaired neuronal homeostasis (Bouget et al., 2025; Müller et al., 2025a). Persistent peripheral immune activation and endothelial dysfunction may disrupt BBB integrity and promote inflammatory signaling within the central nervous system (Greene et al., 2024). Experimental studies have demonstrated that inflammatory cytokines and oxidative stress can activate microglia and astrocytes, leading to sustained production of pro-inflammatory mediators, altered synaptic regulation, and mitochondrial dysfunction (Teleanu et al., 2022; Bouget et al., 2025; Müller et al., 2025b; Pacca-Corrêa et al., 2026).

Experimental studies using murine models, together with complementary investigations in human fetal tissues, have demonstrated that CMV exhibits preferential tropism and persistence within the hippocampus and gray matter of the central nervous system. More recently, neuron-restricted CMV latency has been shown to be actively maintained by tissue-specific immune surveillance mediated by CD4^+^ T cells and IFN-γ, highlighting the importance of local immune regulation in preserving viral latency within the CNS. These findings suggest that age-related deterioration of neuroimmune resilience, together with disruption of blood–brain barrier integrity and chronic systemic inflammation, may compromise these protective mechanisms, thereby increasing susceptibility to neuroinflammation and cognitive dysfunction during aging and long COVID (Tsutsui et al., 2005; Krstanovic et al., 2025).

CMV-associated immune activation has additionally been linked to accelerated immunosenescence and altered neuroimmune communication, which may contribute to impaired neuronal resilience and cognitive decline during aging. Chronic inflammatory signaling within neural tissues may therefore establish localized neuroinflammatory circuits that reinforce tissue dysfunction and vulnerability to neurodegenerative processes (Müller and Di Benedetto, 2024a; Müller and Di Benedetto, 2026).

In the cardiovascular system, CMV-associated inflammatory remodeling may promote endothelial dysfunction, vascular inflammation, and thrombogenic signaling. Endothelial cells represent important targets for CMV persistence, and experimental studies have shown that CMV infection can induce expression of adhesion molecules, inflammatory cytokines, and procoagulatory factors within vascular tissues. Chronic endothelial activation may enhance leukocyte recruitment, oxidative stress, and vascular stiffness while impairing tissue perfusion and barrier integrity (Müller and Di Benedetto, 2024c). Human studies have associated CMV seropositivity with increased arterial stiffness, vascular inflammation, and elevated cardiovascular risk in older individuals (Rahbar and Soderberg-Naucler, 2005; Walker et al., 2009; Bomfim et al., 2023). In long COVID, where endothelial injury and microvascular dysfunction are already prominent features, latent viral reactivation may further amplify vascular inflammatory circuits and contribute to persistent cardiovascular symptoms (Müller and Di Benedetto, 2023b; Müller and Di Benedetto, 2024a; Müller and Di Benedetto, 2024c; Gaspar et al., 2025).

Peripheral tissues may likewise develop localized inflammaging circuits characterized by fibrosis, metabolic dysfunction, impaired regeneration, and stromal remodeling (Kotsaris et al., 2023; Wang et al., 2024). Chronic inflammatory signaling can disrupt tissue repair processes by altering extracellular matrix organization, fibroblast activity, mitochondrial metabolism, and stem-cell function (Matz et al., 2022). Persistent CMV-associated immune activation may additionally contribute to local oxidative stress and inflammatory cytokine production that reinforce tissue degeneration over time. Such mechanisms may be particularly relevant in metabolically active tissues, including skeletal muscle, liver, and adipose tissue, where chronic inflammatory stress can impair regenerative and metabolic capacity.

The gastrointestinal tract may represent another important site of viral inflammaging circuits. Age-associated dysbiosis, impaired epithelial barrier integrity, and alterations of the gut virome may promote microbial translocation and chronic immune activation, thereby amplifying systemic inflammation. Because mucosal immune networks constitute a major component of host resilience, disruption of gut barrier function may further destabilize host–virus equilibrium and contribute to persistent inflammatory signaling during aging and long COVID (Conway and N, 2021; Chen et al., 2022; Caetano-Silva et al., 2024; Babakhani et al., 2025).

Chronic tissue-specific inflammatory remodeling may also contribute to immune dysregulation associated with autoimmunity and cancer (Herbein, 2018; Gugliesi et al., 2021). Persistent inflammatory activation and prolonged cytokine exposure can disrupt mechanisms of immune tolerance, promote aberrant antigen presentation, and alter local immune-cell composition, thereby creating microenvironments permissive for autoreactive immune responses (Hiemstra et al., 2001; Müller and Di Benedetto, 2023b). Associations between CMV infection and autoimmune diseases, including rheumatoid arthritis and systemic lupus erythematosus, have been reported in human studies, although causality remains incompletely established (Bendiksen et al., 2000; Bassyouni et al., 2019). Proposed mechanisms include molecular mimicry, bystander activation, epitope spreading, and chronic interferon signaling, all of which may contribute to sustained tissue inflammation and immune dysregulation (Gugliesi et al., 2021).

Similarly, chronic viral inflammaging circuits may establish conditions favoring oncogenic permissiveness within aging tissues. Persistent inflammatory signaling, immune exhaustion, mitochondrial dysfunction, and impaired immune surveillance can collectively promote genomic instability, stromal remodeling, angiogenesis, and altered cellular stress adaptation (Soroceanu et al., 2011; Herbein, 2018). Although CMV is not generally considered a classical oncogenic virus, viral nucleic acids and proteins have been detected in several tumor-associated tissues, including glioblastoma and breast cancer (Soroceanu et al., 2011; Taher et al., 2013). Experimental studies further suggest that CMV-associated inflammatory signaling may modulate tumor microenvironments through effects on cytokine production, angiogenic pathways, immune evasion, and metabolic adaptation (Soroceanu and Cobbs, 2011; Herbein, 2018). Within aging organisms characterized by reduced resilience and chronic inflammatory stress, viral inflammaging circuits may therefore contribute indirectly to tissue environments that facilitate tumor progression and degenerative pathology.

## 5 Systems gerovirology perspective

As discussed in the preceding sections, the interactions between aging, latent viral persistence, and chronic inflammatory remodeling are highly multidimensional and cannot be fully explained through reductionist single-pathway models. Instead, these processes likely emerge from dynamic interactions among immune, metabolic, epigenetic, and tissue-specific regulatory networks operating across multiple biological scales. In this context, a *systems gerovirology* perspective may provide a useful conceptual framework for understanding how age-associated decline in organismal resilience influences host–virus relationships and contributes to chronic disease vulnerability.

Although the present framework focuses on CMV as a paradigmatic latent virus, future systems-gerovirology approaches should also investigate how endogenous viral elements, including human endogenous retroviruses (HERVs), interact with host resilience networks and contribute to inflammatory and epigenetic remodeling during aging and long COVID (Censi et al., 2024; Guo et al., 2024).

Geroscience has emphasized that hallmarks of aging, including mitochondrial dysfunction, genomic instability, epigenetic alterations, cellular senescence, and chronic inflammation, are deeply interconnected rather than independent pathological processes (Kennedy et al., 2014). Similarly, systems virology approaches increasingly recognize that latent viruses interact extensively with host signaling networks, immune pathways, and cellular stress responses throughout the lifespan (Law et al., 2013; Grossman et al., 2022). Persistent viruses such as CMV therefore can be viewed as long-term biological modifiers capable of reshaping immune architecture, metabolic regulation, and tissue homeostasis.

Within this framework, host resilience may be considered an emergent systems property reflecting the capacity of interconnected biological networks to maintain functional stability under conditions of stress (Müller and Di Benedetto, 2026). Aging-associated deterioration of these networks may progressively reduce resilience thresholds, increasing susceptibility to viral reactivation, inflammatory amplification, and tissue dysfunction (Grossman et al., 2022; Lopez-Otin et al., 2023; Müller et al., 2026b). Importantly, resilience decline is unlikely to occur uniformly across individuals or tissues. Instead, heterogeneity in immune history, metabolic state, genetic background, environmental exposures, and latent viral burden may influence how host–virus equilibrium is maintained or disrupted over time.

The concepts of *host–virus resilience networks* and *viral inflammaging circuits* may therefore help integrate diverse observations across aging biology, immunology, and post-viral syndromes into a unified systems-level model. In this model, CMV persistence interacts bidirectionally with aging-associated inflammatory and metabolic processes, contributing to network destabilization that may ultimately promote multisystem dysfunction. Long COVID may represent a clinically relevant example of this phenomenon, in which persistent immune activation and metabolic stress intersect with latent viral biology to accelerate resilience decline.

## 6 Future directions and conclusions

Despite increasing recognition of the relationship between aging, chronic inflammation, and latent viral persistence, many fundamental questions regarding the mechanisms governing host–virus homeostasis remain unresolved. In particular, it is still unclear why some individuals maintain stable control of latent CMV throughout life, whereas others develop reactivation-prone inflammatory states associated with tissue dysfunction and chronic disease vulnerability. Addressing these questions will require moving beyond reductionist models focused on isolated pathways or individual biomarkers toward integrative approaches capable of capturing the dynamic and multidimensional nature of host–virus interactions.

Future studies should aim to better characterize the molecular and cellular determinants of resilience networks that preserve viral latency during aging and physiological stress. Longitudinal investigations integrating immune profiling, metabolic analyses, epigenetic characterization, and systems-level computational modeling may help identify early signatures of resilience decline and latent viral destabilization. Greater attention should also be given to tissue-specific host–virus interactions, particularly within the nervous, vascular, and immune systems, where chronic inflammatory remodeling may have important functional consequences. Because long COVID shares multiple biological features with accelerated aging, including immune dysregulation, mitochondrial dysfunction, and endothelial stress, this condition may provide a valuable clinical framework for studying how resilience breakdown contributes to latent viral reactivation and chronic inflammatory disease progression.

Importantly, the viral inflammaging circuit proposed here should be regarded as a conceptual and testable systems-level model rather than an established mechanistic pathway. While individual components—including CMV latency, immunosenescence, mitochondrial dysfunction, chronic inflammation, and endothelial dysfunction—are supported by substantial experimental and clinical evidence, their integration into self-reinforcing feedback circuits remains a hypothesis that requires validation through longitudinal, multi-omics, and systems biology approaches.

Beyond exogenous latent viruses, future systems-gerovirology studies should also explore how endogenous viral elements, including human endogenous retroviruses including HERVs, interact with host–virus resilience networks and contribute to inflammatory, metabolic, and epigenetic remodeling during aging and long COVID.

Thus, future research will likely benefit from integrative approaches combining systems immunology, multi-omics technologies, longitudinal aging studies, and network-based modeling to better characterize the mechanisms governing host–virus homeostasis. Single-cell transcriptomics, epigenetic profiling, spatial omics, and computational network analyses may provide important insights into tissue-specific resilience states and inflammatory feedback loops associated with latent viral persistence. Such approaches may ultimately help identify biomarkers of resilience decline and clarify why some individuals remain capable of maintaining stable viral latency despite aging or chronic physiological stress, whereas others transition toward reactivation-prone inflammatory states.

From a translational perspective, identification of key regulatory nodes within host–virus resilience networks may enable the development of “circuit-breaking” interventions targeting senescent cells, chronic inflammation, immunometabolic dysfunction, or viral reactivation. Future studies integrating systems gerovirology with precision medicine approaches may help identify combinations of antiviral, metabolic, and immune-modulating therapies capable of restoring host resilience rather than targeting isolated pathogenic pathways.

In conclusion, the concepts proposed in this review further highlight the need for greater integration between geroscience, systems immunology, and virology. Rather than considering latent viral reactivation solely as a secondary consequence of immune decline, it may be more appropriate to view persistent viruses as dynamic participants in aging biology that interact continuously with host regulatory systems. Within this framework, *host–virus resilience networks* represent the interconnected mechanisms that maintain viral latency and physiological stability, whereas *viral inflammaging circuits* describe self-reinforcing feedback loops linking inflammation, viral activity, tissue stress, and progressive resilience erosion. CMV persistence may serve as a model for understanding how chronic host–virus interactions shape biological aging and post-viral disease states. A *systems gerovirology* perspective may therefore provide a broader conceptual foundation for understanding how latent viral persistence contributes to chronic inflammatory and degenerative processes across the lifespan.
